# Personality disorders and violence: what is the link?

**DOI:** 10.1186/s40479-015-0033-x

**Published:** 2015-09-17

**Authors:** Richard Howard

**Affiliations:** Institute of Mental Health, University of Nottingham Innovation Park, Jubilee Campus, Triumph Road, Nottingham, NG7 2TU UK

**Keywords:** Violence, Personality disorder, Psychopathy, Impulsiveness, Emotion dysregulation, Delusions

## Abstract

Despite a well-documented association between personality disorders (PDs) and violence, the relationship between them is complicated by the high comorbidity of mental disorders, the heterogeneity of violence (particularly in regard to its motivation), and differing views regarding the way PDs are conceptualised and measured. In particular, it remains unclear whether there is a causal relationship between PDs and violence, and what the psychological mechanisms might be that mediate such a relationship. Here, a perspective on PD and violence is offered that views the relationship between them through the lenses of the Five Factor Model of personality and a quadripartite typology of violence. Evidence is reviewed suggesting that emotion dysregulation/impulsiveness, psychopathy, and delusional ideation conjointly contribute to the increased risk of violence shown by people with PD, and do so by contributing to a broad severity dimension of personality dysfunction. This view is consistent with the abandonment of personality disorder categories in the forthcoming eleventh edition of the International Classification of Diseases (ICD-11), where severity of personality disorder is defined in terms of the degree of harm to self and others.

## Introduction

### Aims and objectives

This review aims to cast light on the relationship between personality disorders (PDs) and violence, in particular on the possible mechanisms that mediate the relationship. While the literature clearly indicates that PDs are linked to violence [1, 2], an answer to the question “What is the link?” is obscured by several factors. First, confusion exists over how PDs should best be conceptualised and assessed, confusion that is exacerbated by the high degree of comorbidity that exists between different PDs [3], making their boundaries fuzzy (see Different perspectives on personality disorder: types or traits? section below). Second, confusion exists over how best to classify violence, given its heterogeneity (see Heterogeneity of violence section below). Third, any attempt to infer a causal relationship between PD and violence is fraught with difficulties. Given the overwhelming co-occurrence of multiple disorders, particularly in forensic psychiatric patients, it becomes extremely difficult to specify what is responsible for the link with violence. Moreover, violence has been found to be linked to a range of mental disorders in addition to PD [1], including schizophrenia (OR 7.4) [4]), bipolar disorder (OR 5.8) [5]), and depression (OR 3.0) [6]. In reviewing the functional link between PD and violence, Duggan and Howard [2] gave the example of a patient who meets criteria for both narcissistic and paranoid PDs, has multiple (DSM-IV) Axis I conditions including substance use and post-traumatic stress disorder (PTSD) and is prone to violence. They ask: “Does one give precedence to a blow to the individual’s self-esteem (narcissism), or to a suspiciousness of the motives of others (paranoid traits), his or her substance abuse or the activation of PTSD symptoms in explaining his or her violent behaviour?” (p.25). In other words, which condition has causal primacy in determining the links between mental disorder and violent behaviour? Below it will be suggested that to understand the link between PD and violence, one needs to move beyond traditional diagnostic categories to a consideration of trans-diagnostic variables such those identified in a New Zealand birth cohort followed prospectively into adulthood [7]. In this study, psychiatric disorders were initially explained by three higher-order factors, Internalising, Externalising and Thought Disorder, but were explained even better by one general psychopathology factor (p), with thought disorder symptoms at its pinnacle. This general psychopathology factor was suggested to represent overall severity of psychopathology.

The central argument in this paper is first, that consideration of the motivational heterogeneity of violence requires a typology of violence that can empirically account for the varieties of violence encountered in those deemed mentally disordered. Details of such a typology, and its empirical support, are detailed under Heterogeneity of Violence below. The second contention is that the propensity to violence is linked not just to severity of personality disorder, but to overall level or severity of psychiatric morbidity. In general, the greater the degree of psychiatric morbidity (and, it is argued, general psychopathology or p), the higher is the risk of violence. It is further suggested that this broad dimension of PD severity is underpinned by emotional impulsiveness, psychopathy in its various guises, and delusional ideation. Evidence is reviewed below under Personality Disorder and Violence that supports this contention.

### Different perspectives on personality disorder: types or traits?

Prior to the emergence of DSM-5 in 2013 [8], it was a longstanding criticism of the diagnostic categories for PD contained in previous editions of the DSM that they lacked specificity or discriminant validity; that is to say, there was considerable overlap between PD categories. As a consequence, different PDs frequently co-occurred in the same individual; indeed, high PD comorbidity is more often the rule than the exception [3]. Unlike in general community and clinical samples, comorbidity between antisocial and borderline PDs has been found to be especially prevalent in forensic psychiatric samples, reaching nearly 80 % in women deemed “dangerous and severely personality disordered” [9]. This highlights the lack of generalizability of PD comorbidity patterns from one type of sample to another [3]. Moreover, antisocial/borderline PD comorbidity has been found to be strongly associated with degree of severe violence perpetrated by personality disordered offenders [10]. Over half of the comorbidity between antisocial and borderline PDs is reported to arise from genetic factors [11], and these disorders share genetic and environmental risk factors over and above those common to all Cluster B disorders (antisocial, borderline, narcissistic and histrionic) [12].

The Five Factor Model (FFM) of personality can help us understand why some PDs are more highly comorbid with each other than others [3]. In particular, it explains why antisocial PD is most highly associated with borderline PD. In terms of FFM, both antisocial PD and borderline PD are primarily characterized by low levels of Agreeableness facets and low levels of Conscientiousness facets, but in addition borderline PD is significantly and positively related to all Neuroticism facets (anxiousness, angry hostility, depressiveness, self-consciousness, impulsiveness and vulnerability). These considerations were largely the impetus for development of the trait-based approach adopted in section 3 of DSM-5, where 5 domains or dimensions of personality - broadly aligned with the FFM domains - are represented: Antagonism, Disinhibition, Psychoticism, Negative Affectivity and Detachment, each comprising a constellation of more specific traits. These traits are assessed using the Personality Inventory for Diagnostic and Statistical Manual of Mental disorders (PID-5 [13]).

However, adoption of a trait-based approach has evidently not solved the problem of a lack of discriminant validity in relation to PD diagnosis. A recent study [14] reporting correlations between PID-5 traits and measures of FFM personality domains found, firstly, that there were high correlations across the DSM-5 personality domains, indicating a high degree of cross-domain overlap. Secondly, specific traits from 4 of the 5 DSM-5 domains correlated significantly and positively with FFM Neuroticism (the exception was Antagonism), while traits from all 5 DSM-5 domains correlated significantly and negatively with both Agreeableness and Conscientiousness. Of note, the general psychopathology (‘p’) factor identified in a New Zealand birth cohort followed up into adulthood showed a positive association with Neuroticism and inverse associations with both Agreeableness and Conscientiousness [7]. Crego and colleagues point out: “To the extent that a p factor is the explanation for the current findings, one might then in turn suggest that perhaps the longstanding criticism of the weak discriminant validity for the DSM-1 V-TR personality disorders…..has to some degree been overstated or misunderstood” ([14], p.12). Another study [15] that developed a bi-factor model of PD traits identified a general (g) factor of PD that transcended diagnostic boundaries. The g factor appeared to index overall PD severity and to represent a mixture of antisocial PD traits (irresponsible, disregard for safety, failure to conform, deceitfulness, impulsivity), traits related to cognitive disturbance (odd beliefs, ideas of reference), and traits related to internalising/neurotic introversion (socially inhibited, avoids social contacts at work, preoccupied with rejection), as well as traits related to obsessionality. Other factors identified traits related to specific PDs, with the exception of borderline PD traits which loaded only on the g factor. This is not surprising considering that, of all PDs, BPD is the only one that includes symptoms of dysfunction across all four domains of cognition, affectivity, interpersonal behaviour and impulse control [16].

Another recent factor-analytic study [17] identified two higher-order psychopathology factors, Externalising and Internalising, and correlated these with 30 FFM personality facets. The higher-order Internalising factor was positively associated with all facets of Neuroticism and negatively associated with two facets of Extraversion, positive emotions and assertiveness. The higher-order Externalizing factor was positively associated with two FFM facets of Neuroticism (angry hostility and impulsivity) and with three facets of Extraversion (excitement seeking, gregariousness and activity). It was negatively associated with all facets of Conscientiousness and with three facets of Agreeableness: straightforwardness, compliance and modesty. These results suggest that, in keeping with the argument above, antisocial/borderline PD comorbidity can be seen as reflecting the combination of high Externalising and high Internalising. Those in whom antisocial PD co-occurs with borderline PD will show, by virtue of high Externalizing, exceptionally high levels of angry hostility, impulsivity and excitement seeking, together with traits reflecting low Conscientiousness and low Agreeableness. By virtue of high Internalizing they will additionally show very high levels of traits associated with Neuroticism and low levels of some traits related to Extraversion (particularly a lack of positive emotions) and Conscientiousness (particularly low competence and lack of self-determination). Evidence to be reviewed below suggests that this combination of Externalising and Internalising traits is associated with severely violent offending in personality disordered offenders. We first need to consider how violence is best conceptualised.

### Heterogeneity of violence

**Fig. 1:**
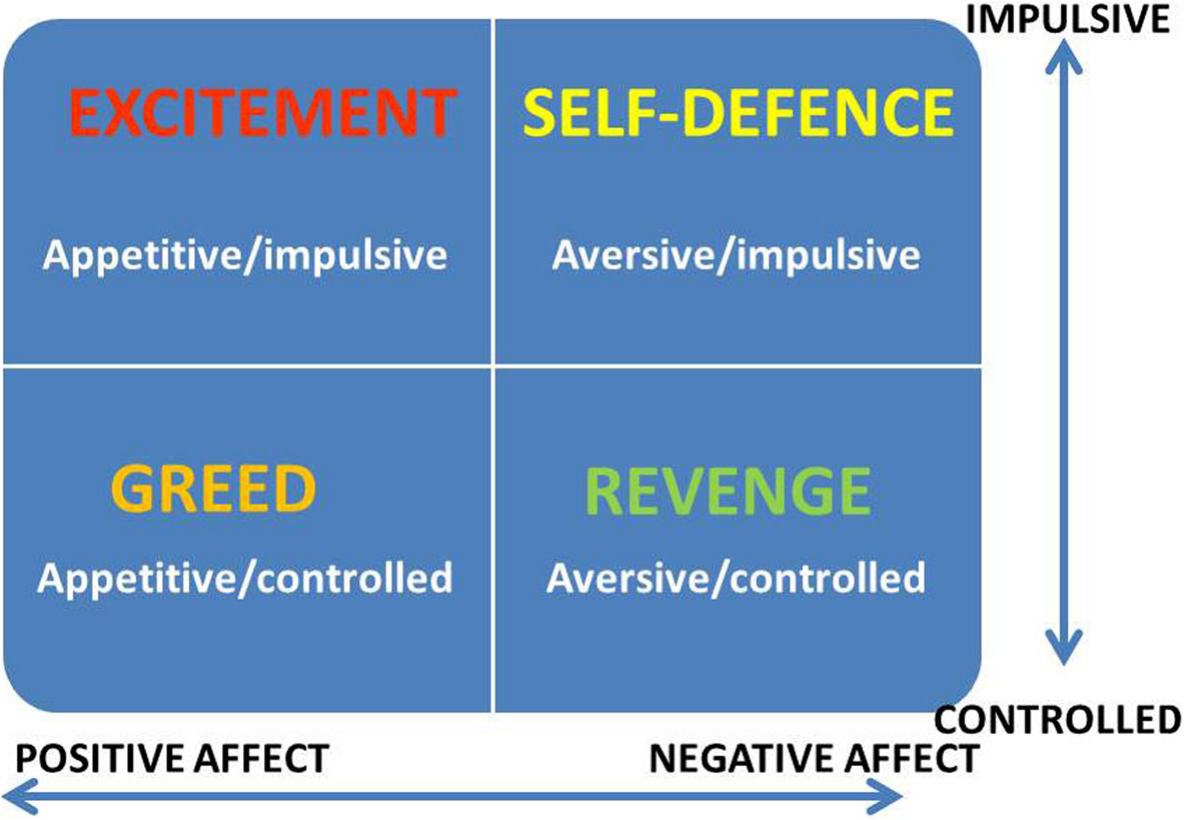
The quadripartite (2×2) violence typology. The intersection of impulsiveness (vs. control/premeditation) and affect (positive vs. negative) yields 4 distinct types of violence characterised by motives of excitement seeking and greed (both associated with positive affect) and revenge and self-defence (both associated with negative affect)

The heterogeneity of violence is another problem that obscures its relationship with mental disorders in general, and PD in particular. Violence is a complex phenomenon that varies with regard to victims, severity, frequency and context, and comprises distinct types. Traditional typological distinctions, e.g., proactive/instrumental vs. reactive aggression and impulsive vs. premeditated, although often used interchangeably, represent overlapping but distinct constructs that, as reviewed in [18], are conceptually and empirically distinct and may have different aetiologies. These authors state that “new models of aggression that can account for the shared and unique characteristics of impulsive and proactive aggression are in order “([18], p.259). One such new model is the quadripartite violence typology (QVT) proposed by the current author [9, 19] whose development was in large part driven by the perceived inability of the traditional reactive/instrumental dichotomy to accommodate all forms of violence, including appetitive violence. According to QVT, an act of violence may be either impulsive or controlled/premeditated and, within each of these categories, is either appetitively or aversively motivated. This yields the four violence types shown in Fig. 1, each associated with the achievement of a particular goal: enhancement of positive affect through infliction of suffering on others in the case of impulsive/appetitive violence (upper left quadrant in Fig. 1); reduction of negative affect through removal of an interpersonal threat in the case of of impulsive/aversive violence (upper right quadrant in Fig. 1); gaining of material goods or social dominance in the case of controlled/appetitive violence (lower left quadrant in Fig. 1); and retribution for some perceived slight or grievance in the case of controlled/aversive violence (lower right quadrant in Fig. 1). Each type of violence is associated with a particular affective state (positive or negative) and a particular constellation of emotions: fear and distress in the case of aversively motivated violence carried out impulsively; spite and vengefulness in the case of aversively motivated violence carried out in a controlled way; exhilaration and excitement in the case of appetitively motivated violence carried out impulsively; and pleasant anticipation in the case of appetitively motivated violence carried out in a controlled way. All four violence types are said to involve dysregulated emotions, with violence associated with either an excess of positive emotions (e.g., exhilaration or greed in the case of appetitive violence) or an excess of negative emotions (e.g., fear/distress or resentment/vengefulness in the case of aversive violence).

While retaining the traditional distinction between impulsive and premeditated aggression, QVT incorporates the distinction between reactive (impulsive/aversive type in Fig. 1) and proactive/instrumental (controlled/appetitive type in Fig. 1). QVT allows for a far richer representation of the motivations that drive violence in those who are mentally disordered. Using a list of functions of violence previously identified [20], QVT was validated in a sample of violent young men by showing that a unique set of functions predicted each of the of the four types shown in Fig. 1 [21]. For example, the appetitive/impulsive’ violence type was uniquely and positively predicted by the combination of functions labelled ‘sensation seeking’ and ‘observe suffering’. Further validation was provided in a study of Australian youths who had been convicted of a violent offence [22]. QVT allowed for adequate classification, in an uncomplicated manner, of all violent offences and proved superior to the traditional reactive/instrumental classification. In both studies, thrill-seeking was found to be strongly associated with violent offending in a proportion of delinquent youths, supporting the idea that the quest for excitement can be a powerful motivation for violence in some (perhaps mostly male) young offenders. In female young offenders, revenge or retribution has been reported as a major motivation for violence [23]. Interestingly, in this study revenge-motivated violence was classified as “instrumental”, in contrast to QVT where it is classed as controlled/aversive (lower right quadrant in Fig. 1) rather than controlled/appetitive. In QVT the latter is reserved for violence motivated by a desire for material gain or social dominance.

### Is there a causal relationship between PD and violence?

The finding of an association between PD and violence, reviewed below, does not necessarily imply a causal connection between them. Establishing causality requires that three criteria be met in addition to the co-variation between PD and violence [2]. First, PD must occur prior to the violent offending. This issue of temporal precedence is problematic since it requires that the developmental emergence of PD occurs prior to the emergence of violent offending; moreover, that developmental trajectories can be specified according to which a history of violent offending emerges in the context of a prior PD. However, contemporary views of PDs (e.g., borderline PD [24, 25]) emphasise their early development in childhood and adolescence. The forthcoming ICD-11 revision will not exclude a diagnosis of PD on the grounds of age: “personality disorder most commonly has its first manifestations in childhood and is clearly evident in adolescence” ([26], p.722). It is therefore reasonable to view violence emerging out of, or in the context of, a developmentally prior PD. Two possible pathways from childhood and adolescence to adult violence are outlined below under Separate Pathways from Internalizing and Externalizing to Violence.

Second, alternative explanations for the relationship must be excluded - the possibility of third variables, of which a number exist, e.g., abuse of alcohol and other psychotropic drugs as precipitants of violence, must be eliminated. This is important to consider given evidence that alcohol misuse is an important causal risk factor for the commission of violent crimes in young men [27], particularly crimes involving impulsive acts of assault [28].

Lastly, and most importantly, a causal mechanism linking PD with violence must be specified in order to address the question of how PD causes violence. This is the most difficult and challenging task facing those interested in elucidating the relationship between PD and violence, and three likely candidates in this regard are reviewed below. Are there mechanisms specific to each disorder, e.g., delusions for psychosis, impulsiveness for personality disorders, mania for bipolar disorder? This is unlikely given the evidence reviewed above for general psychopathology factors such as Externalizing and Internalizing that transcend traditional diagnostic boundaries. Evidence is reviewed below to support the contention that the link between PD and violence is best explained by three critical mechanisms - emotional impulsiveness, psychopathy, and delusional ideation - that are associated with the combination of these higher-order factors. The review is deliberately selective, focusing on intrapersonal factors, and does not exclude the possibility of many other (e.g., psychosocial) factors being involved in the aetiology of violence. The focus here is on recent studies that have related PD to violence.

## Review: personality disorder and violence

### Sub-groups of PD in relation to violence

While an association between PD and violence has been well documented [1, 2], the literature highlights the importance of considering particular sub-groups of patients with PD that are characterised by particular patterns of comorbidity and gender. For example, results of a meta-regression analysis [1] emphasised that the relationship between PD and violence is not straightforward and varies by PD category and by gender. Thus while the overall OR for PD was 3.0, the risk of violence was highest for those with antisocial PD, particularly in women (OR 13.1) compared with men (OR 7.9). In non-forensic clinical samples, co-occurrence of antisocial and borderline PDs is seen more frequently in men than in women [29, 30]. However, in forensic samples, particularly those at the high-severe end of the PD spectrum (e.g., women classified as having “dangerous and severe PD”), antisocial PD comorbid with borderline PD occurs more commonly in women than in men [9]. The higher risk of violence in women with antisocial PD compared with men is therefore likely accounted for by their showing a higher co-occurrence of borderline PD. This can only be surmised, however, since the above-mentioned meta-regression analysis [1] did not examine comorbidity of PDs in relation to violence.

Subsequent research has confirmed the importance of examining subgroups of PD individuals characterised by particular patterns of comorbidity, as well as gender and ethnicity. A recent study of violence perpetrated by American prison inmates, both male and female and of black and white ethnicity, reported that regardless of ethnicity, those with co-occurring psychopathy and antisocial PD were almost twice as likely, compared with other inmates, to have a history of severe and versatile violent offending [31]. Violent offending was highest in black males and females with comorbid antisocial PD and psychopathy, pointing to the importance of both gender and ethnicity in addition to PD comorbidity in rates of violence among offenders. In patients recruited as part of the McArthur study, borderline PD with co-occurring psychopathic traits was associated with violence during a one-year study period [32]. A triple comorbidity – antisocial PD with co-occurring borderline PD and psychopathy – was found to be associated with the highest rates of severe violent offending among men diagnosed with PD and detained in medium or high security in the UK [10]. The co-occurrence of antisocial personality and borderline PD in a UK household sample was significantly associated with a history of violence, but this was largely, although not entirely, accounted for by co-occurring alcohol dependence, anxiety disorder and severe childhood conduct disorder (CD) [33]. A study that compared non-violent men with violent men who were, or were not, gang members, reported very high levels of psychiatric morbidity (with the exception of depression) in both the latter groups but particularly in gang members [34]. Compared with non-violent men, violent men who were not gang members were more likely to show psychosis (OR 2.9), anxiety (OR 1.8), alcohol dependence (OR 1.6) and antisocial PD (OR 8.8), and to have made use of psychiatric services (ORs 1.9 –2.7). Equivalent ORs for gang members, who showed the highest level of violence, were 4.2, 2.2, 6.5, 57 and 4.3–7.8. A large proportion of violent men who were gang members reported being excited by violence (63 %) and using violence instrumentally (73 %), suggesting that in terms of the quadripartite violence typology (Fig. 1), gang members’ violence was most often of the impulsive/appetitive type.

In short, while a clear relationship appears to exist between personality disorder (e.g., antisocial PD when this co-occurs with other PDs, and particularly with borderline PD) and violent offending in general, rates of violent offending differ by degree of psychiatric morbidity, by gender and by ethnicity. In general, the greater the psychiatric morbidity and comorbidity, the greater is the risk of violence. This suggests that risk of violence may be related to overall severity of psychopathology (p).

### Impulsiveness and dysregulated affect

#### Impulsiveness

Impulsiveness can broadly be defined as a predisposition to react rapidly and without planning to internal and external stimuli with lack of regard for short-term and long-term consequences for oneself and others [35]. It is considered to be a symptom of many psychiatric disorders including borderline and antisocial PDs, bipolar disorder, attention deficit/hyperactivity disorder, conduct disorder and substance abuse/dependence. Although impulsiveness has been commonly assumed to be linked to violence, this link is questionable, particularly in psychosis where comorbidity is a significant issue [35, 36].

The variable findings in the field are likely accounted for by the heterogeneity of both violence and impulsiveness. First, as discussed above, not all violence is impulsive, and not all impulsive violence is motivated in the same way (see Fig. 1). Secondly, impulsiveness is multifaceted, incorporating a number of dimensions, including a tendency to act rashly and intemperately under the pressure of positive or negative emotions [37]. When behaving in an emotionally impulsive way, the individual responds to a stimulus or event on the basis of an immediate emotional reaction such as desire or anger, with little if any checking of long-term consequences [38]. It is apparent that impulsive violence as defined in the typology outlined in Fig. 1 is related specifically to emotional impulsiveness rather than to other aspects of impulsiveness such as a tendency to think or act rashly. Measures of impulsiveness, both self-report and behavioural, are limited in the degree to which they tap emotional impulsiveness. For example, a commonly used self-report measure of impulsiveness, the Barratt Impulsivity Scale (BIS) [39], does not include an explicitly emotional component.

#### The UPPS model and measures of impulsive behaviour

The UPPS is “a promising measure and model of impulsivity because it conceptualises and assesses impulsivity as a multifaceted construct that includes various, separable, and distinct pathways to impulsive behaviour….” ([40] p.4). UPPS includes a scale, negative Urgency, which reflects “a tendency to experience strong impulses, frequently under conditions of negative affect” ([41] p. 685). Subsequently UPPS was revised to include a positive Urgency scale to reflect impulsive behaviour occurring in the context of positive affect. Positive and negative Urgency were found to correlate highly and were considered as a unitary scale in a study that examined relationships between UPPS scales (Urgency, [lack of] Perseverence, [lack of] Premeditation, and Sensation Seeking), and DSM-5 PDs assessed both categorically (DSM-5 section 2) and by traits (DSM-5 section 3) [40]. Urgency correlated most strongly with PD traits – with 3 of the 5 trait domains (Negative Affectivity, Antagonism and Disinhibition), and with 14 of 25 lower-order traits. This lack of discriminant validity suggests that, rather than reflecting specific PD types, Urgency reflects overall PD severity. Both Urgency and (lack of) Premeditation correlated with antisocial and borderline PDs, while Sensation Seeking correlated only with Histrionic and Narcissistic PDs. The authors suggested that (lack of) Premeditation and Urgency may help explain the high rates of externalising behaviours associated with antisocial and borderline PDs. Supporting this, it was found that the incidence of serious physical violence committed by psychiatric inpatients was increased threefold in those who scored high on Urgency, and was nearly two times higher in those with PD (specific types of PD were not examined in this study) [42]. Another study [10] found that a composite measure of serious violence comprising serious violence in the criminal record, early onset of violent behaviour and serious institutional violence correlated significantly with both Urgency and (lack of) Premeditation. A subsequent closer examination of the data from this study (unpublished observations, Howard and Khalifa) showed that Urgency correlated significantly (*p* < .01) and positively with dimensional scores of 5 out of 10 PD categories (paranoid, antisocial, borderline, histrionic and dependent) and with overall PD severity defined by the combination of internalizing and externalizing PD traits. Exceptionally, Urgency correlated significantly (*p* < .05) and *negatively* with schizoid PD. Unlike antisocial PD, which was significantly associated with all UPPS facets, borderline PD did not correlate significantly with Sensation Seeking. This suggests that borderline PD is distinguishable from antisocial PD in lacking the excitement seeking facet of Externalizing that characterises antisocial PD [17].

#### Emotion dysregulation

Emotion dysregulation, also known as affective instability or emotional lability, is a key feature of borderline PD and is a lower-order trait within the domain of Negative Affectivity in the DSM-5 section 3 trait-based typology. However, the high overlap beween DSM-5 domains has been noted above, and high and significant correlations were reported between emotional lability and many traits across other domains [14]. Affective instability overlaps both conceptually and empirically with affective impulsiveness or Urgency [40] and correlates significantly and positively with FFM Neuroticism and negatively with both Agreeableness and Conscientiousness [14]. Evidence suggests that emotion dysregulation mediates the link between borderline PD and violence [43, 44]. The latter study found a relationship between emotional dysregulation and perpetration of physical assault that was mediated by emotion dysregulation but not by trait impulsiveness. Notably, however, trait impulsiveness was measured using an FFM-based metric that predominantly tapped non-emotional facets of impulsiveness such as self-discipline and deliberation.

It may be concluded from these studies that emotional impulsiveness – the tendency to respond rashly and to act out under pressure of high emotional arousal - is the facet of impulsiveness that is most relevant to the link between PD (particularly borderline PD) and violence. However, it appears likely that emotional impulsiveness contributes importantly to overall PD severity. Therefore it is considered likely that it is PD severity, rather than emotional impulsiveness or emotion dysregulation *per se*, that accounts for the high degree of violence associated with borderline PD. Small wonder, then, that a physiological correlate of emotional impulsiveness was successful in identifying those mentally disordered offenders who were at high risk of re-offending violently upon their release from secure care into the community [45]. Evidence that this physiological correlate is changeable, and is therefore a dynamic rather than static risk factor, offers hope that intervention pre-release can potentially reduce the risk of violent re-offending [46].

### Psychopathy

Psychopathy as conceptualised and measured by the currently most used instrument, the Psychopathy Checklist (PCL) is a much-debated construct [47] and is not accepted as a valid psychiatric condition by all forensic psychiatrists (e.g., [48]). Nonetheless, it is commonly regarded as a personality disorder and has entered the nomenclature of DSM-5 under the label “Antisocial/Psychopathic Personality Disorder” [8] where, in section 3, a psychopathic features specifier has been included to designate the classically low-anxious, socially assertive variant of antisocial personality described in the adult psychopathy literature [49–52].

The most recent, 20-item revision of the PCL (PCL-R) consists largely of items assessing dispositions such as impulsiveness and behaviours such as pathological lying and criminal versatility [53]. It yields, in addition to a total score, scores on two factors – selfish, callous and remorseless use of others (F1), and chronically unstable and antisocial lifestyle (F2). Each factor subsumes two facets, so that F1 subsumes the facets Interpersonal and Affective, while F2 subsumes the facets Lifestyle and Antisocial. Some authors have warned against reliance on the total PCL-R score to “diagnose” psychopathy. According to Lilienfeld and colleagues reliance on total PCL-R scores “…is no longer defensible given that the subdimensions of most psychopathy measures are associated with substantially different personality correlates” ([54], p.30). Howard & Duggan suggested that given the high heterogeneity within the class of high PCL-R scorers, “a high score on the PCL may tell us little more about the individual than that he or she is a high PCL scorer – one may just as well call him or her ‘a bastard’..” ([55], p. 284). It is not surprising, therefore, that the PCL-R factors are differentially related to violence, with Factor 2 showing a considerably stronger relationship than Factor 1, as reviewed in [55]. These authors conclude: “…. it appears to be the combination of criminal behaviour and poor behavioural controls (irritability, aggression and inadequate control of anger) that, among traits tapped by the PCL, accurately predicts future violent and non‐violent offending” (p.284).

Studies concur in showing that PCL-R psychopathy maps onto normal personality traits measured using the FFM, and that central to it is (lack of) Agreeableness. PCL psychopathy appears to be largely an admixture of low Agreeableness and low Conscientiousness, with varying contributions made to the PCL-R factors by Neuroticism and Extroversion [54, 56]. Similarly, psychopathy appears to be embedded within, and to extend across, several categories of PD, in particular antisocial and narcissistic [57, 58]. We have noted above the relationship of the general psychopathology factor (p) with low Agreeableness, low Conscientiousness and high Neuroticism. We must consider therefore the possibility that this particularly toxic combination of personality traits reflects overall severity of psychopathology, and that “psychopathy”, rather than being a unitary disorder, represents a variable constellation of traits that contribute importantly to overall severity of psychopathology. Supporting this, PCL-R scores were reported to be significantly associated with a measure of overall PD severity, obtained by summing across individual PD criteria [59]. A measure of psychopathy derived from an assessment of PD, labelled “acting out” [60], also correlated with PD severity and, in regression analysis, predicted a high degree of severe violence in the criminal records of personality disordered offenders [59].

Given the heterogeneity of violence outlined above, we must still ask whether particular variants, or components, of psychopathy might contribute to different types of violence. Unfortunately, studies looking at violence through the lens of QVT in different subtypes of psychopath are still to be done. Evidence reviewed above suggests that violence to satisfy a lust for excitement is common in delinquent youth and in violent gang members, 86 % of whom qualified for antisocial PD [34]. Narcissistic PD has been reported to be strongly related to causing pain and suffering to others, and this relationship was significant even when other Cluster B personality disorders were controlled [61]. However, the motivation here is unclear, and case reports of men with narcissistic PD suggest their violence is triggered by a slight or insult and is motivated by a desire for vengeance (see for example Case 4 in [62]). In terms of the violence typology shown in Fig. 1, this would clearly correspond to the controlled/aversive violence type, but this type of violence may be more characteristic of the vulnerable narcissist than the grandiose narcissist. While both grandiose and vulnerable sub-types are characterized by low FFM Agreeableness, the vulnerable subtype is additionally associated with prominent Neuroticism traits (e.g., shame, need for admiration) and low Extraversion, while the grandiose sub-type is associated with high Agentic Extraversion (e.g., exhibitionism, authoritativeness); the DSM construct of narcissistic PD captures a mixture of these two sub-types [63]. Borderline and antisocial features of PD may be closely linked to aversively motivated violence, both in its controlled, premeditated form where the motivation is revenge, and in its impulsive form where it is motivated by removal of an immediate interpersonal threat. Violence when it (rarely) occurs in the classic Cleckleyan [49] manifestation of psychopathy may be associated with avarice – greed for material objects or social dominance.

### Delusional ideation

#### Delusional ideation in PD patients

Despite deficits in the cognitive domain being a core area of deficit in the PDs, delusional thinking has been relatively neglected as a possible mediator of violence in personality disorders, notwithstanding its well-documented presence in PD. In borderline PD a “quasi-psychotic” thought disturbance is common, for example a delusional belief in imminent abandonment by a romantic partner or health professional [64]. Recent evidence suggests that delusional ideation is related to overall severity, rather than type, of PD, and that severe violence in forensic patients is related to both severity of PD and degree of delusional thinking [59]. This is consistent with evidence that severity of PD is related to metacognitive deficits that include an impaired ability to recognize the subjective nature of one’s thoughts and to achieve a critical distance when considering one’s beliefs [65]. This would necessarily result in idiosyncratic interpretations of external reality, and would likely result in the types of deficit in social cognition seen in BPD patients, namely: a tendency to misinterpret neutral situations, to feel socially rejected during normative inclusion conditions, and to have difficulty restoring cooperation after experiencing disappointment [66]. A bias towards interpreting neutral or ambiguous social encounters as threatening, as demonstrated for example in [67], would impact negatively on borderline patients’ everyday social interactions and predispose them to react to interpersonal stress with aggression and violence. They would be particularly susceptible to the impulsive/aversive type of violence (Fig. 1) that is associated with interpersonal threat.

Recent evidence suggests that delusions implying threat or harm to the individual are associated with angry affect, and that angry affect due to delusions mediates the latter’s relationship with serious violence [68]. It therefore seems likely that angry affect resulting from threat-related delusional thinking, and in particular the inability to regulate that affect, may be a critical link in the pathway leading from psychosis to serious violence. The degree of conviction with which delusions are held may be a factor in determining the emotional response [35].

#### Separate pathways from internalizing and externalizing to violence

As noted above, a study of forensic psychiatric patients with PD found a relationship between severity of delusional ideation and severe violence [59]. Regression analysis revealed that severe violence was predicted by high scores on two trans-diagnostic dimensions of PD, “acting out” (equivalent to psychopathy) and “anxious-inhibited” (equivalent to neurotic introversion). However, while “acting out” independently predicted severe violence, the effect of “anxious-inhibited” on violence appeared to be mediated by delusional ideation. This result mirrors those from the New Zealand cohort study [7] referred to above, where a general psychopathology factor (p) linked to delusional thinking and an Externalizing factor emerged as independently associated with violent offending – both correlated significantly and positively with violent conviction. Internalizing was inversely associated with violence when p was taken into account. Externalizing was positively associated with FFM Extraversion but not associated with Neuroticism, while p was strongly associated with Neuroticism and inversely associated with Agreeableness and Conscientiousness.

Taken together, these results suggest that separate developmental pathways might link Externalising and Thought Disorder aspects of PD with violence. One pathway, operating via delusional thinking and general psychopathology (p), would result from extremely high and/or inflexible levels of negative emotionality, empathy and rumination experienced in the context of intense and/or chronic interpersonal stress during adolescence, particularly in females [69]. Another, externalising pathway would lead from severe conduct disorder in early childhood, particularly when this co-occurs with callous-unemotional traits [70], to adult antisocial personality, via effects of alcohol misuse in early adolescence on adolescent brain development [10, 71, 72]. These pathways would not, of course, be mutually exclusive. Indeed, both pathways would be expected to operate conjunctively in those with an end-point of severe PD (e.g., those with co-occurring antisocial and borderline PD).

## Conclusions

**Fig. 2:**
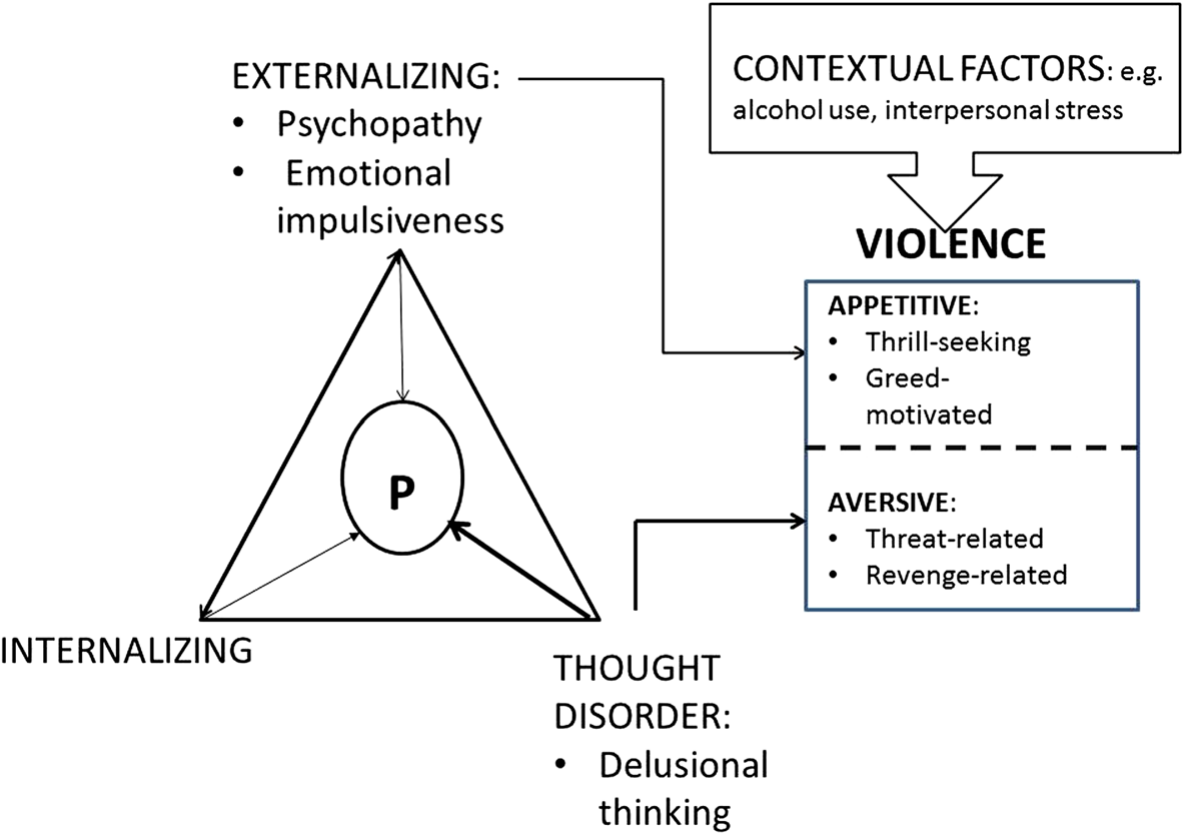
A schematic overview of the suggested relationship between personality disorder (PD) and violence. General psychopathology (p) is subsumed by 3 factors, Externalizing, Internalizing and Thought Disorder but, as reported in [7], p is associated most strongly with Thought Disorder (indicated by a heavy arrow in the figure). Externalizing subsumes traits associated with both psychopathy and emotional impulsiveness, both of which contribute to severe PD and increase the risk for violence, particularly appetitive violence. Thought Disorder is shown as contributing independently to the risk of violence, and particularly of aversive violence. Also shown are contextual factors such as alcohol use that operate as proximal causal risk factors for violence in concert with distal personality factors

Viewing the PD/violence relationship through the lenses of the FFM on the one hand, and of QVT on the other hand, permits a new perspective on the relationship between PD and violence. This perspective suggests that a higher-order Externalizing dimension, subsuming both emotional impulsiveness and “psychopathy” in its various guises, together with Thought Disorder, can account for the relationship between PD and violence: see Fig. 2. In general, the higher the prevalence of traits associated with *both* Thought Disorder *and* Externalizing, the greater would be the severity of PD and hence the greater the propensity for violence. Note that the relationship between Internalizing and Externalizing is shown as bidirectional in Fig. 2, since internalising and externalising tendencies are known to co-occur in adolescents and that this co-occurrence was found to be mediated by rumination in adolescent males [73]. Nonetheless, different combinations of traits related to Thought Disorder and Externalizing would likely be associated with different manifestations of violence, specifically with differences in the four different types of violence proposed by QVT: thrill-seeking (motivated by a desire for excitement), threat-related (motivated by a desire to remove a perceived interpersonal threat), revenge-related (motivated by a desire for vengeance), and greed-related (motivated by a desire for material goods or social dominance). Although empirical verification is currently lacking, It would be expected, for example, that those showing high levels of Externalising traits, resulting in symptomatology characterised by irresponsibility, manipulation, wilfulness and both cognitive and behavioural dysregulation [17], would be biased towards showing appetitive (thrill-seeking and greed-related) forms of violence (Fig. 2). Thrill- seeking violence should be more prominently seen in those scoring high on the excitement seeking facet of Extraversion and low on facets of Agreeableness. Those showing high levels of disordered thinking, on the other hand, would be biased towards showing aversively motivated (threat-related and revenge-related) forms of violence, as shown in Fig. 2. The co-occurrence of traits related to both Externalizing and Thought Disorder seen in patients with borderline/antisocial PD comorbidity, particularly in female patients with this comorbidity, constitutes a particularly toxic concatenation of traits that would be expected to be associated with a high risk of severe violence that is both appetitively (e.g., thrill-seeking) and aversively (e.g., threat-related) motivated.

It is perhaps appropriate that the forthcoming edition of the International Classification of Diseases (ICD-11) will abandon the previous personality disorder typology (apart from the presence of personality disorder itself, which is largely defined as a dysfunction of interpersonal behaviour), in favour of a classification according to level of severity [26]. Here severity is defined by the degree of harm to self and others, ranging from mild (“not associated with substantial harm to self or others”) to severe (“associated with a past history and future expectation of severe harm to self or others that has caused long-term damage or has endangered life” ([26], p. 722). Now that personality disorder will no longer be defined by questionable types, but by the degree of harm done, maybe the focus of research can switch to a closer examination of the various motivations underlying violence associated with different permutations of dysfunctional trait domains, listed in ICD-11 under the headings Negative Affect, Dissocial, Disinhibition, Anankastic and Detachment. It is hoped that the quadripartite typology outlined in Fig. 1 will help in this respect, although other typologies exist and should also be considered [74].
